# Single-cell genomics-based analysis reveals a vital ecological role of *Thiocapsa* sp. LSW in the meromictic Lake Shunet, Siberia

**DOI:** 10.1099/mgen.0.000712

**Published:** 2021-12-03

**Authors:** Yu-Ting Wu, Pei-Wen Chiang, Kshitij Tandon, Denis Yu Rogozin, Andrey G. Degermendzhy, Sen-Lin Tang

**Affiliations:** ^1^​ Department of Forestry, National Pingtung University of Science and Technology, Pingtung 91201, Taiwan, ROC; ^2^​ Biodiversity Research Center, Academia Sinica, Taipei 115, Taiwan, ROC; ^3^​ Institute of Biophysics, Siberian Division of the Russian Academy of Sciences, Krasnoyarsk, Russia; ^4^​ Siberian Federal University, Krasnoyarsk, Russia

**Keywords:** flow cytometry, Lake Shunet, purple sulfur bacteria, single-cell genomics

## Abstract

Meromictic lakes usually harbour certain prevailing anoxygenic phototrophic bacteria in their anoxic zone, such as the purple sulfur bacterium (PSB) *Thiocapsa* sp. LSW (hereafter LSW) in Lake Shunet, Siberia. PSBs have been suggested to play a vital role in carbon, nitrogen and sulfur cycling at the oxic–anoxic interface of stratified lakes; however, the ecological significance of PSBs in the lake remains poorly understood. In this study, we explored the potential ecological role of LSW using a deep-sequencing analysis of single-cell genomics associated with flow cytometry. An approximately 2.7 Mb draft genome was obtained based on the co-assembly of five single-cell genomes. LSW might grow photolithoautotrophically and could play putative roles not only as a carbon fixer and diazotroph, but also as a sulfate reducer/oxidizer in the lake. This study provides insights into the potential ecological role of *Thiocapsa* sp. in meromictic lakes.

## Data Summary

fastq sequences have been deposited in the National Center for Biotechnology Information Sequence Read Archive under the BioProject accession number PRJNA252899.

**Impact Statement**

Lake Shunet is one of the four meromictic lakes located in Siberia. Meromictic lakes usually harbour specific prevailing anoxygenic phototrophic bacteria in their anoxic zone, such as the purple sulfur bacterium (PSB) *Thiocapsa* sp. LSW (hereafter LSW) in Lake Shunet. PSBs have been indicated to play a key role in element cycling at the chemocline of stratified lakes; however, the ecological significance of PSBs in the lake remains poorly understood. A deep-sequencing analysis of single-cell genomics associated with flow cytometry was used to explore the potential ecological role of LSW. Based on the approximately 2.7 Mb draft genome, LSW might grow photolithoautotrophically and could play putative roles not only as a carbon fixer and diazotroph, but also as a sulfate reducer/oxidizer in the lake. This study provides insights into the potential ecological role of *Thiocapsa* sp. in meromictic lakes.

## Introduction

Researchers have recently shown an increased interest in meromictic lakes, which have layers of water that do not intermix and comprise specific zones with stratified and unique biogeochemical characteristics [1–5]. As a consequence of the layers, such lakes usually harbour uncommon microbial communities [2–4, 6, 7], specifically prevailing anoxygenic phototrophic bacteria (i.e. purple sulfur bacteria; PSBs), such as *Lamprocystis purpurea* in Lake Mahoney [8], *Chromatium okenii* in Lake Cadagno [9], *Rheinheimera* sp. in Lake Shira [6], *Halochromatium* sp. in Lake Oigon [6], *Thiocapsa* sp. in Lake Shunet [6, 7] and *Thiocapsa marina* in Lake Trekhtsvetnoe [10]. Many studies have suggested that PSBs play an important role in the cycling of various nutrients, including carbon, nitrogen and sulfur, specifically at the oxic–anoxic interface of stratified lakes [9, 11–19]; however, the ecological role of PSBs in Lake Shunet remains unclear.

Lake Shunet (54° 25' N, 90° 13' E), located in the North-Minusinsk Depression of the central Altai-Sayan Mountain region in southern Siberia, is one of the only four meromictic lakes in the entire Asian region of Russia [20]. The lake is shaped by the local climate (evaporation) and water resource (mainly snow), which give it sharp gradients in salt content; parts of Lake Shunet have high salinity (~71 g l^-1^) and hydrogen sulfide levels (~400 mg H2S l^-1^) in the monimolimnion, and a dense layer of PSB species known as *Thiocapsa* sp. in the interface zone [6, 7, 21], which is different from other meromictic lakes, such as Lakes Shira, Cadagno and Ace [22–24]. *Thiocapsa* has previously been described from brackish coastal, marine environments and freshwater [25–27]; the *Thiocapsa* sp. LSW (hereafter LSW, for Lake Shunet Water) from Lake Shunet was the first *Thiocapsa* described from an inland saline meromictic lake. *Thiocapsa* spp. are anoxygenic phototrophic PSBs that use hydrogen sulfide, thiosulfate, elemental sulfur and molecular hydrogen as electron donors during photolithotrophic growth [25]. *Thiocapsa* cells have been described as non-motile, cocci-shaped bacteria [25, 27, 28]. However, the ecological role of *Thiocapsa* in this specific lake ecosystem is not well described. As most microorganisms in the field are difficult to cultivate [29], the single-cell genomic approach based on whole-genome amplification has become widely used in microbial ecology. A single cell represents a distinct informational entity for an organism [30], making the approach particularly useful for investigating the ecology of microbial species or individual isolates that are difficult to cultivate [31–34].

The only genome-centric study of *Thiocapsa* was carried out by Hemp and colleagues [31], who isolated and cultured the genus from a sewage-treatment facility. Cultivation-based methods might obscure the microorganisms that are difficult to cultivate in a complex microbial community, so we aimed to isolate single cells directly from lake water using flow cytometry [35], then use the single-cell genomics technique to clarify the ecological importance of the prevalent *Thiocapsa* sp. of Lake Shunet in Siberia. Therefore, we provide comprehensive insights into the potential ecological role of *Thiocapsa* sp. in this lake based on a genome-centric analysis. Furthermore, based on the percentage of read counts in bins that were binned from metagenomes of Lake Shunet, *Enterobacter* sp. was identified as another prevailing microbial group in the chemocline, along with *Thiocapsa* sp. [7]; thus, we explored the potential interactions between *Enterobacter* sp. and *Thiocapsa* sp. in this specific niche using genome annotation.

## Methods

### Sample collection

Water samples for single-cell sorting were collected at 5.0, 5.1 and 5.2 m deep with a multi-syringe stratification sampler [36] on July 21 2010 from Lake Shunet (54° 25' N, 90° 13' E) in the Republic of Khakasia, Siberia. The entire sampling time was ~2–2.5 h, including the time it took to transport the samples back to the field station at Shira. Water samples were immediately filtered through a 10 µm plankton net and then divided into Falcon 50 ml conical centrifuge tubes. The samples were then transported in ice bags back to Academia Sinica (Taipei, Taiwan). There, they were divided into aliquots and stored at −80 °C until further processing.

The sampling process for determining the bacterial community composition and metagenomes was different from single-cell sorting. Nearly 20 l of water samples was subsequently filtered through a 10 µm plankton net and placed in a Millipore-Pellicon TFF system (0.22 µm filter membrane) to collect microbes on the membranes in the local field station at Lake Shira [7].

### Single-cell sorting, whole-genome amplification and PCR screening

Water samples were prepared for single-cell sorting as follows. Each sample (approximately 300 µl) was thawed on ice and centrifuged at 12 000 ***g*** for 10 min, the supernatant was removed, and cells were washed three times with 300 µl 1× PBS. Then, cells were resuspended in 1 ml 1× PBS and immediately subjected to a fluorescence-activated MoFlo XDP cell sorter (Beckman Coulter) with a PE-Cy 7 detector as the main trigger requirement. Specifically, we first compared the scatter plot SSC (side scatter) to the FSC (forward scatter) based on the particle size and internal granularity, and then the emission in the infrared portion of the spectrum. The region that included the LSW population (R1) was selected and one cell was deposited into one PCR tube containing 5 µl TE buffer (Fig. S1, available with the online version of this article). Single cells were handled and downstream PCR was processed under the most stringent conditions to avoid contamination.

We used a procedure for lysing fluorescence-activated sorted single cells modified from that of Siegl *et al*. [37], including two cycles of thawing and freezing (70 and −80 °C, respectively; 10 min each), followed by the addition of 5 µl denaturation buffer (0.4 M KOH, 10 mM EDTA) and incubation at 65 °C for 3 min. Then, 0.5 µl neutralization buffer (1×0.8 M Tris-HCl, pH4) was added. Whole genome amplification was performed in a total volume of 50 µl based on phi29 polymerase-mediated multiple displacement amplification using the REPLI-g mini kit (Qiagen). Whole genome amplification was initially incubated at 30 °C for 10–16 h, then the phi 29 DNA polymerase was inactivated by heating the sample for 3 min at 65 °C. The amplified genomic DNA was stored at −20 °C until further processing.

The amplified single-cell genomic DNAs were screened using Sanger sequencing on the V1–V2 hypervariable regions of the 16S rRNA gene. PCR was carried out in a total volume of 50 µl containing 2.5 U *Superrun EX Taq* HS, 5 µl 10× *EX Taq* buffer, 200 µM dNTPs, 0.2 µM each primer (27F 5′-AGAGTTTGATCMTGGCTCAG-3′ and 341R 5′-CTGCTGCCTCCCGTAGG-3′) [38] and 2–5 µg single-cell multiple displacement amplification products. The PCR programme was as follows: an initial denaturation at 95 °C for 5 min; followed by 30 cycles at 95 °C for 20 s, 52 °C for 20 s and 72 °C for 20 s; and a final extension step at 72 °C for 5 min; then cooling at 4 °C. Six single-cell multiple displacement amplification products were identified as *Thiocapsa* sp. and sent for shotgun sequencing and analysis based on the clean Sanger sequencing electropherogram.

### Genome sequencing, assembly and bioinformatics

We sorted and analysed more than 200 single cells, but only 6 single cells were classified as the same *Thiocapsa* sp. strain based on the 16S rRNA with 99 % identity against the National Center for Biotechnology Information (NCBI) database and with 100 % identity among the six 16S rRNA sequences. The genomes of these six single cells were sent for sequencing and individual single-cell assemblies were initially performed with SPAdes [39] using *k*-mer sizes of 21, 33 and 55. One sample was removed from the genome assembly due to contamination of ambiguous sequences. Furthermore, average nucleotide identity (ANI) was calculated one versus the other among the five single cells. Contigs ≥1000 bp were used for ANI calculations using http://enve-omics.ce.gatech.edu/ani/index [40]. All ANI values ranged from 99 to 100 % (Table S1). Therefore, the current draft genome was co-assembled using reads from five single cells.

Briefly, 2 µg of five individual single-cell amplified DNAs were sheared, then purification, end-repairing and ligation were performed. The library was sequenced using the Illumina HiSeq 2000 sequencing platform with the mode 2×150 bp at Yourgene Bioscience (Taiwan). The single-cell reads obtained were quality checked with FastQC (https://www.bioinformatics.babraham.ac.uk/projects/fastqc/), quality filtered with a minimum phred score of 20 and adapter trimmed using AdapterRemoval [41]. Reads with ambiguous bases were also removed. Quality filtered, non-ambiguous and trimmed reads were co-assembled with megahit [42] using *k*-mer sizes of 21, 33 and 55. Contigs obtained from co-assembly were subjected to automatic binning with concoct [43] with minimum contig size set to 1000 bp. concoct uses nucleotide *k*-mer frequencies and coverage data from multiple samples to perform an unsupervised binning. Binning was conducted to remove any contaminant sequences that the kit might have held from the data. Bins obtained from concoct were then checked for their completeness and contamination using CheckM [44]. Bins with completeness >50 % and contamination ~1 % were selected for downstream analysis. Gene prediction was performed with Prokka, which uses Barrnap [45] (https://github.com/tseemann/barrnap) as the default for 16S rRNA gene prediction. ORFs were annotated by searching against the EggNOG (http://eggnog.embl.de) and the Kyoto Encyclopedia of Genes and Genomes [46] databases using blastp (*E* value ≤10 ^− 5^ and bit score ≥100).

### Phylogenetic analysis

Available 16S rRNA gene sequences for *Thiocapsa* species were downloaded from the NCBI Taxonomy database (last accessed August 2020). We selected 35 sequences, including one 16S rRNA gene sequence of *Thiobaca trueperi* as the outgroup, for phylogenetic tree reconstruction. Sequence alignment was performed with cmalign from the infernal package [47], which performs a covariance model (CM)-guided small subunit rRNA alignment. The CM for the bacterial domain was downloaded from the rfam database (last accessed January 2020) [48]. The alignment was subjected to web-based Gblocks [49] to extract conserved blocks with default settings. A maximum-likelihood phylogenetic tree (with 914 bases) was computed using iq-tree v1.6.11 [50] with the TN+F+I model – selected based on Bayesian information criterion (BIC) values – and 1000 bootstraps. The tree obtained was visualized and edited in iTOL v4 [51].

In addition, a phylogenetic tree based on single-copy marker proteins was also reconstructed. Sequenced genomes of four *Thiocapsa* (* Thiocapsa* sp. UBA6158, *Thiocapsa roseopersicina* DSM 217, *Thiocapsa marina* 5811 and *Thiocapsa rosea* DSM235) and *Thiohalocapsa marina* DSM 19078 and *Thiocystis violascens* were downloaded from the NCBI Genomes database and a phylogeny was reconstructed using single-copy marker proteins and a two-step approach. Single-copy marker proteins were identified and aligned using ezTree [52]. The alignment obtained from ezTree was used to reconstruct a maximum-likelihood phylogeny with iq-tree with the JTTDCMut +F+I+G4 model – selected based on BIC values – and 1000 bootstraps. A consensus tree was visualized and edited in iTOL v4.

## Results

### Genome properties and phylogeny

More than 18 million high-quality reads were obtained and assembled into 563 contigs with 127× coverage. The assembly of the LSW single-cell genome resulted in a total size of 2.7 Mb (Table 1). The draft genome was about 50.39 % complete with 1.12 % contamination based on the CheckM results; therefore, the LSW was assumed to be approximately 5.4 Mb long.

**Table 1.** Properties of the single-cell genome of *Thiocapsa* sp. LSW

Assembly	* Thiocapsa* sp.
**Raw reads (paired end**)	
WGA1-9	1 906 273×2
WGA11-10	2 024 697×2
WGA12-4	2 850 582×2
WGA12-6	2 121 645×2
WGA12-16	1 950 558×2
**QC filtered and trimmed reads (paired end**)	
WGA1-9	1 879 220×2
WGA11-10	1 969 896×2
WGA12-4	2 764 148×2
WGA12-6	2 058 986×2
WGA12-16	1 884 446×2
**Assembly statistics**	
No. of contigs	563
Genome size (bases)	2 707 436
No. of genes	2656
No. of coding sequence	2626
No. of rRNA	4 (5S, 16S, 23S)
No. of tRNA	25

**Fig. 1.**
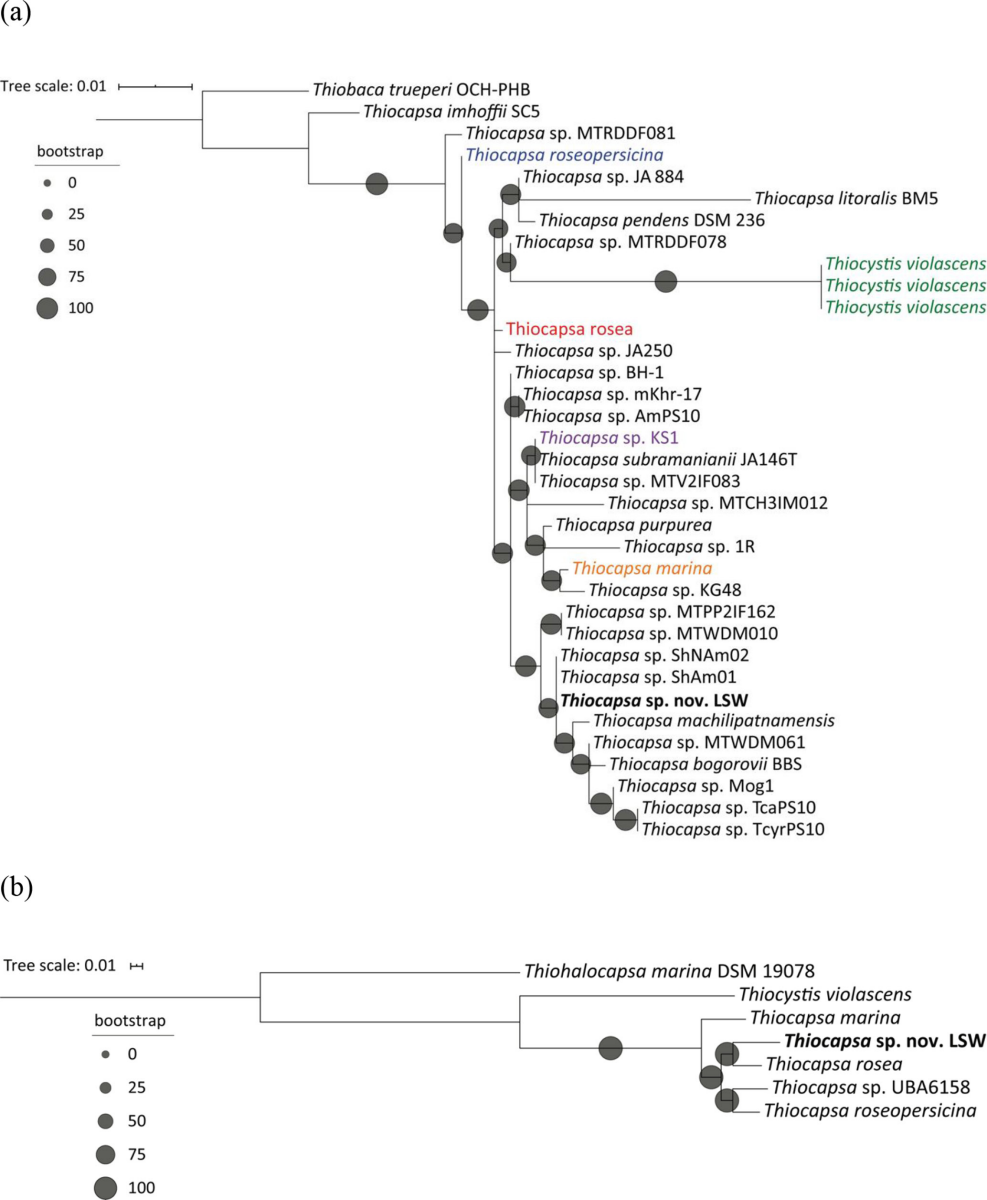
Phylogenetic analyses of *Thiocapsa* sp. LSW based on (**a**) 16S rRNA genes, the sequences of which were downloaded from the NCBI Taxonomy database, with *Thiobaca trueperi* as the outgroup; (**b**) 248 single-copy marker genes. Sequenced genomes of *Thiocapsa* spp. were downloaded from the NCBI Genomes database. Both trees were reconstructed using the maximum-likelihood method with 1000 bootstraps in iq-tree, and visualized and formatted in iTOL. *Thiocapsa* with coloured text in (**a**) were also analysed for single-copy marker genes in (**b**).

One copy of the 16S rRNA gene was annotated in this draft genome, and a phylogenetic tree was built based on the 16S rRNA gene and the other three available *Thiocapsa *, three *Thiocystis violascens* and one *Thiobaca trueperi* 16S rRNA genes from the NCBI Taxonomy database (Fig. 1a). The phylogenetic tree shows that LSW is closely related to *Thiocapsa* sp. ShAm01 and ShNAm02, which were isolated from Lake Shira and Lake Shunet, respectively. A heatmap was constructed based on the 16S rRNA gene identity (%) between the closest relatives of *Thiocapsa* (Figs 1a and S2). In addition, 248 single-copy marker proteins (Table S2) were identified in the five available genomes of *Thiocapsa* (including *Thiocapsa* sp. LSW from this study), and a phylogenetic tree based on single-copy marker proteins was reconstructed (Fig. 1b). LSW from this study was phylogenetically close to *Thiocapsa rosea *.

### Phototrophy

**Fig. 2.**
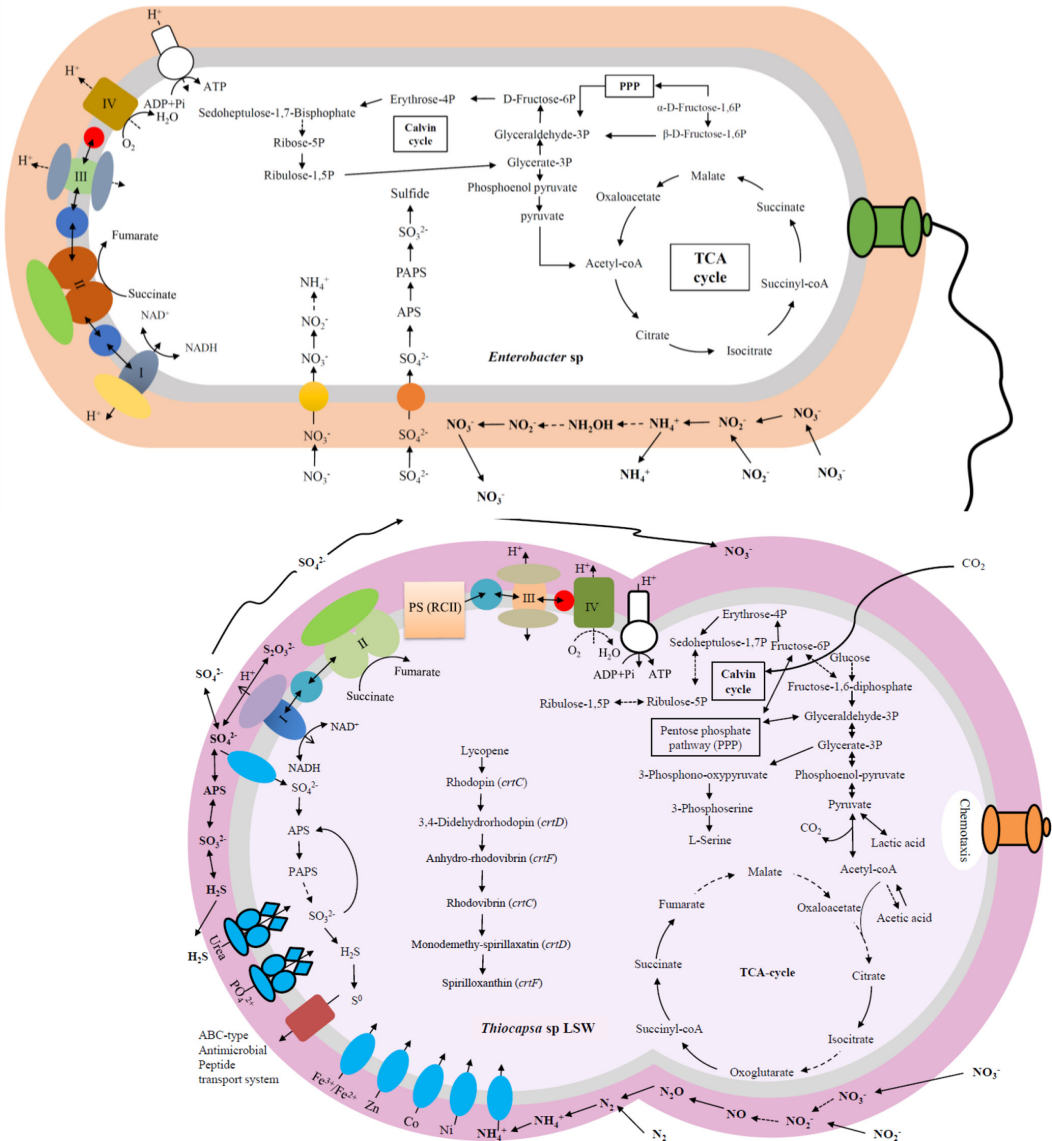
Putative interactions between the two most prevalent groups in the chemocline layer in Lake Shunet: *Thiocapsa* sp. LSW (shown with a purple outermost layer) and *Enterobacter* sp. (shown with an orange outermost layer). The dotted line with the arrow denotes the genes that were not annotated in the draft genome.

Only one gene (*pufC*; cytochrome c subunit) involved in the RCII gene cluster of LSW was annotated, and it had 97 % sequence identity to the closest sequenced strain, *Thiocapsa rosea* DSM 235. Multiple copies of genes (*pufA* and *pufB*) for the main antenna of purple bacteria (light-harvesting complex LHI) and photosynthetic reaction centre cytochrome c subunit (*pufC*) were also present. PucC is a subunit involved in the photosynthetic reaction centre that encodes RC-LHI auxiliary proteins, which are membrane-bound bacteriochlorophyll (BChl) synthesis enzymes. In addition, two copies of high-potential iron-sulfur proteins (HiPIPs), which generate an electrochemical potential coupled to ATP synthesis, were annotated. The genes related to the pigments that were detected in their entirety were required for the biosynthesis of spirilloxanthin, which might be the major carotenoid in LSW, with the exception of BChl (Fig. 2).

### Carbon metabolism

LSW might undergo autotrophy due to CO_2_ assimilation via the Calvin–Benson–Bassham cycle, which is nearly completely annotated in the draft genome. Two copies of *rbcL* and *rbcS* genes encoding ribulose-bisphosphate carboxylase (type I RuBisCO) large and small subunits were present. Moreover, genes encoding phosphoenolpyruvate carboxylase for phosphoenolpyruvate carboxylation involved in the C4-dicarboxylic acid cycle were also annotated. Carboxysome shell proteins and carbonic anhydrase for the conversion of bicarbonate to carbon dioxide in carboxysomes were also detected.

### Nitrogen metabolism

**Fig. 3.**
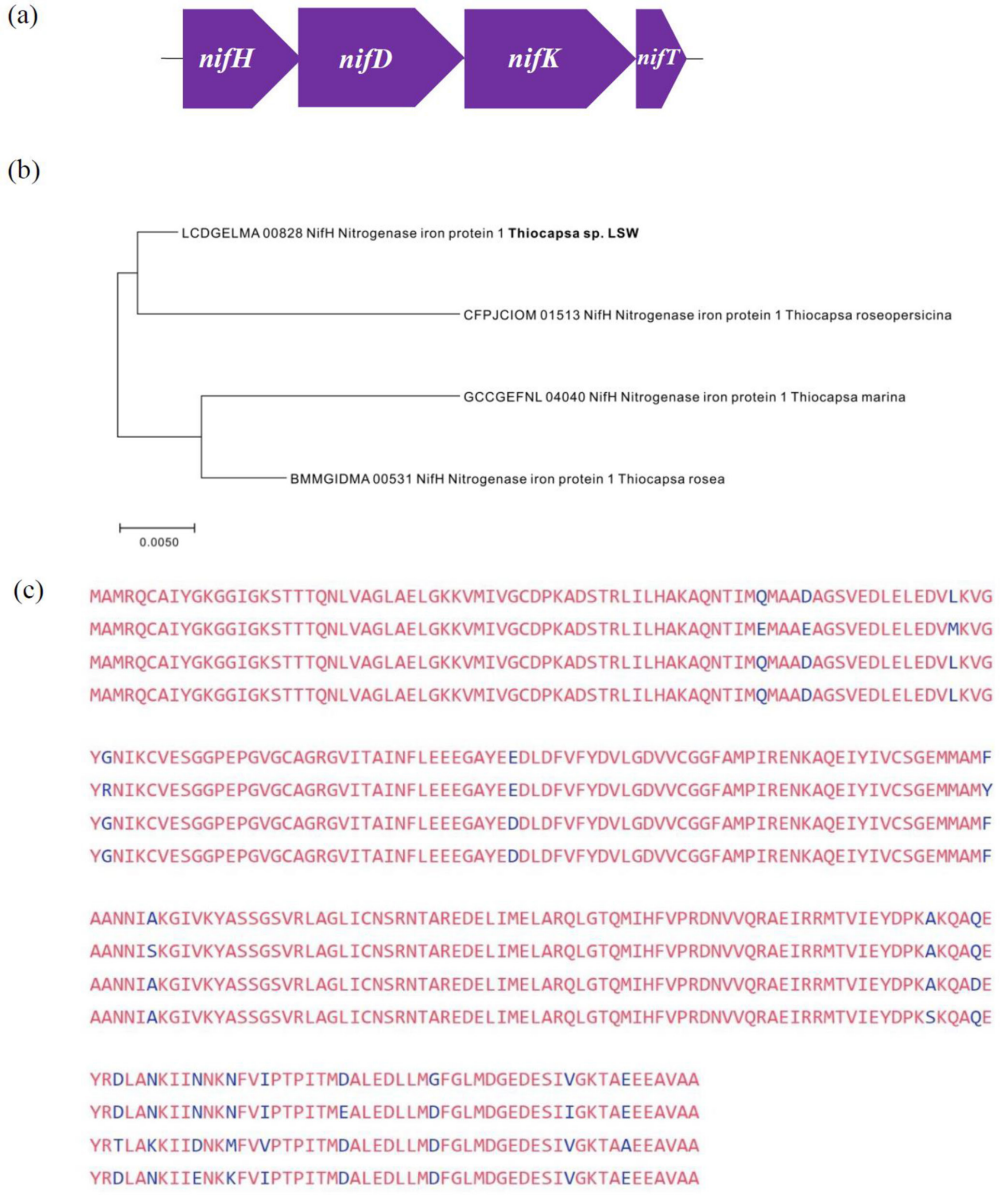
Nitrogen fixation. (**a**) Schematic diagram of the complete *nifHDKT* operon of LSW and three *Thiocapsa* species. (**b**) Phylogenetic tree of *Thiocapsa* species based on the *nifH* gene and reconstructed using the maximum-likelihood method with 1000 bootstraps. (**c**) Alignment of *nifH* amino acid sequence of *Thiocapsa* species (from the top down, LSW, *Thiocapsa roseopersicina *, *Thiocapsa marina* and *Thiocapsa rosea *). Identical amino acids are highlighted in red; different amino acids are highlighted in blue.

A complete set of *nif* genes for nitrogen fixation was annotated, including molybdenum-iron nitrogenase (NifDK), nitrogenase-stabilizing/protective protein (NifW) and nitrogenase reductase (NifH), suggesting that LSW acts as a diazotroph in Lake Shunet. Specifically, the complete *nifHDKT* operons of four *Thiocapsa* spp. including LSW were annotated, and the phylogenetic tree reconstructed based on *nifH* protein sequences (294 aa) indicated that LSW was functionally related to *Thiocapsa roseopersicina* in terms of nitrogen fixation (Fig. 3). Although no genes involved in assimilatory nitrate or nitrite reduction were annotated in the current draft genome, a gene encoding the nitrate/nitrite transporter (NRT) was detected. Therefore, LSW might utilize nitrate or nitrite as nitrogen sources. In addition, the presence of genes encoding urease (*ureABC*) and urea transport system permease (*urtB*) indicated another potential alternative mechanism by which the organism can obtain nitrogen. LSW had one candidate gene involved in the nitrification pathway: hydroxylamine dehydrogenase (*hao*); these and the potential nitrate/nitrite transporter suggest that the LSW might be able to perform complete nitrification. Four genes related to (NiFe) hydrogenases, which play a role in electron transport, were also present. Generally, LSW can utilize a range of organic and inorganic low-potential electron donors to conserve energy.

### Sulfur metabolism

A wide variety of enzymes involved in sulfur metabolism were annotated in the draft genome of LSW, including those for assimilatory sulfate reduction, dissimilatory sulfate reduction and oxidation, and thiosulfate oxidation. In the oxidation systems, sulfur compounds are utilized as electron donors for anoxygenic photosynthesis. In the reduction systems, a nearly complete assimilatory sulfate reduction pathway via adenosine phosphosulfate, 3′-phosphoadenosine phosphosulfate was annotated, with elemental sulfur (S^0^) as the final product (Fig. 2), which might be stored in cellular sulfur globules. In terms of dissimilatory sulfate reduction, LSW may be capable of conducting the reduction via adenosine phosphosulfate and sulfite, and use sulfate as a terminal electron acceptor to produce H_2_S. In further phylogenetic analyses based on key genes, the presence of *dsrA* for the sulfate reduction and *soxB* for the sulfate oxidation of five *Thiocapsa* spp. suggests that LSW is related to *Thiocapsa marina* and *Thiocapsa rosea *, respectively (Fig. S3).

## Discussion

### Unique *Thiocapsa* sp. from the saline meromictic Lake Shunet

According to the NCBI Taxonomy database, about 31 *Thiocapsa* spp. have been described to date. They are ubiquitous in fresh, marine, alkaline, acidic and hot or cold waters, and include the isolates *Thiocapsa roseopersicina* BBS [53] from the White Sea, *Thiocapsa marina* 5811 from the Mediterranean Sea [25], *Thiocapsa imhoffii* from haloalkaline Soap Lake [54] and *Thiocapsa* KS1 from a sewage-treatment facility [27]. LSW from this study was isolated from the saline meromictic Lake Shunet and clustered independently with the isolates of *Thiocapsa* sp. ShNAm01 and ShAm01 (96 % bootstrap) from Lake Shunet [28] and Lake Shira [55], respectively, in the reconstructed 16S rRNA gene phylogenetic tree. Isolates of *Thiocapsa* spp. from different geographical locations or ecosystems seem to differ based on specific environmental factors and phylogenetics. Furthermore, a genome tree based on 248 single-copy marker genes was built from five available genomes of *Thiocapsa *: *Thiocapsa marina* 5811, *Thiocapsa roseopersicina* DSM217, *Thiocapsa* sp. UBA6158 [56], and *Thiocapsa rosea* DSM235 and LSW (current study). Among these, *Thiocapsa marina* 5811 was isolated from the Mediterranean Sea [25], while *Thiocapsa roseopersicina* DSM217, *Thiocapsa* sp. UBA6158 and *Thiocapsa rosea* DSM235 were isolated from sewage lagoons or wastewater ponds [56–58]. Based on the genome tree, the five *Thiocapsa* genomes represent specific roles in the ecosystems from which they were isolated. LSW represents an independent cluster separate from the other four genomes, indicating that it may have an important ecological role in this saline meromictic lake.

More specifically, the complete *nifHDKT* operon in the nitrogen fixation gene cluster is annotated in LSW, along with three other *Thiocapsa* draft genomes: *Thiocapsa roseopersicina *, *Thiocapsa marina* and *Thiocapsa rosea *. Unlike the phylogenetic analysis of 16S rRNA, LSW clustered instead closely to the type strain, *Thiocapsa roseopersicina *, on the basis of the *nifH* gene. LSW and *Thiocapsa roseopersicina* were from Lake Shunet and a wastewater pond from a sugar mill, respectively; both natural habitats may have common characteristics specifically for nitrogen fixation. This also indicated that phylogenetic analysis based on 16S rRNA and *nifH* genes of *Thiocapsa* showed divergence. The finding was consistent with that of Zehr *et al*. [59], in which both 16S rRNA and *nifH* genes evolved at different rates.

### Potential ecological role of LSW and its interaction with *Enterobacter* sp. in the chemocline

The highest number of phototrophic bacteria was recorded at a depth of 5.0 m; the bacteria included the green sulfur bacteria *Prosthecochloris vibrioformis *, PSBs *Thiocapsa* and *Halochromatium* species, and the purple nonsulfur bacteria *Rhodovulum euryhalinum* and *Pinkicyclus mahoneyensis*, among which *Thiocapsa* was the most dominant phototroph [28]. Our previous study [7] also indicated that the PSB LSW is a major anoxygenic phototrophic bacterium in the anoxic layers of Lake Shunet. Unlike *Thiocapsa marina *, which contains okenone as its main pigment [25], our annotation results suggest that the major carotenoid in LSW is spirilloxanthin, as it is in *Thiocapsa roseopersicina* [60, 61] and *Thiocapsa* KS1 [31].

The photosynthetic apparatus in many purple bacteria – including *Thiocapsa marina* DSM 5653 and *Thiocapsa* KS1 – consists of two light-harvesting antenna complexes (LHI and LHII) [12, 23]; however, only LHI was detected in the current draft genome of LSW. Although no genes responsible for the biosynthesis of BChl were annotated in the current draft genome, we suggest that LSW might utilize BChl α for phototrophic growth. This is because our results used PE-Cy7 for single-cell sorting and detected an emission wavelength in the near-infrared region at about 800 nm, which is within the spectrum characteristic of BChl α in purple bacteria, specifically *Chromatiaceae* [62], and matches the spectral characteristic fluorochromes of PE-Cy7, including near-infrared [63]. This was echoed in the specific absorption spectra of the pigments of *Thiocapsa* in the lake [28].

BChl α has distinct light absorbance from the chlorophyll of cyanobacteria, which might be one of the factors causing *Thiocapsa* sp. and cyanobacteria to colonize densely in the anoxic and oxic zones, respectively. Additionally, the genes encoding enzymes involved in the Calvin cycle were annotated in the draft genome, which supposed that *Thiocapsa* sp. grew photolithoautotrophically in Lake Shunet. This finding agrees with the work by Kondratieva [64], which showed that all *Chromatiaceae* employ the Calvin cycle when undergoing photolithoautotrophic growth. Therefore, LSW was specifically prevalent on the surface of the chemocline due to the light demands for phototrophic development, as there is little doubt that light is vital to LSW in this specific lake. Based on the gene annotation, which identified phosphoenolpyruvate carboxylase and carbonic anhydrases in the draft genome, LSW might enhance carbon fixation efficiency by phosphoenolpyruvate carboxylation and concentrating CO_2_ [65, 66].

In terms of the sulfur cycle, LSW harbours genes involved in the oxidation of sulfide, sulfite and thiosulfate, which might provide electrons to carry out photosynthetic carbon dioxide fixation to produce carbohydrates. This has been experimentally confirmed in anoxygenic phototrophic bacteria including *Chromatium okenii* and *Thiocapsa roseopersicina* BBS [53, 67].

In the metagenomics study, abundance was calculated as the read counts in the bin divided by the total read counts; based on this, *Thiocapsa* sp. and *Enterobacter* sp. (* Enterobacteriaceae *) were the two most abundant bacterial groups in the chemocline of Lake Shunet. The metabolisms conducted by *Enterobacter* sp. were reconstructed (Fig. 2) based on the gene annotation of the assembled genomic bin [7]. As the prevailing bacteria in the chemocline, they might play an important role in nitrogen cycling in the layer, considering that the complete dissimilatory nitrate reduction pathway and partial gene involved in nitrification were annotated (described by Bonin [68]). In terms of the sulfur cycle, only a complete assimilatory sulfate reduction pathway was annotated. The sulfur cycle was reconstructed based on the gene annotation predicted from metagenomes in the chemocline of Lake Shunet, and *Thiocapsa* sp. seemed to be the only bacterium that could undergo dissimilatory sulfate oxidation [7]. The genes involved in the production of SO_4_^2-^ were detected in the draft genome of LSW from this study, which is in accordance with work by Trüper and Fischer [69]. Therefore, LSW might contribute SO_4_^2-^ to other bacteria, including *Enterobacter* sp., which carries out assimilatory sulfate reduction (Fig. 2). In addition, complete genes that encode steps in the nitrification process were annotated in the draft genome of LSW, and LSW likely provides nitrate or nitrite for *Enterobacter* sp. to undergo the nitrate reduction.

A previous metagenomic analysis [7] suggested that *Thiocapsa* sp. oxidizes sulfide in the chemocline and generates sulfite, thiosulfate and sulfate for other microbes in the layer, and the draft genome from the present study confirms this. Therefore, LSW may play a role as both a sulfate reducer and oxidizer in Lake Shunet. In terms of nitrogen metabolism, LSW can undergo denitrification and nitrogen fixation, and preferentially utilizes ammonium as a nitrogen source; this echoes work by Brown and Herbert [70], which showed that ammonium is the preferred nitrogen source for PSBs. Consequently, LSW as a diazotroph in Lake Shunet might also contribute ammonium to other microbes.

## Supplementary Data

Supplementary material 1
