# Supplementary material for: Single-cell genomics-based analysis reveals a vital ecological role of Thiocapsa sp. LSW in the meromictic Lake Shunet, Siberia
Source: Microb Genom. 2021 Dec 3;7(12):000712. doi: 10.1099/mgen.0.000712 (PMC8767323; doi:10.1099/mgen.0.000712)
Supplement: Supplementary material 1 [file mgen-7-0712-s001.pdf]

Table S1 ANI (average nucleotide identity) (%) among the five single cells.

	WGA-11-10	WGA-12-4	WGA-12-6	WGA-1-9	WGA-12-16
WGA-11-10	100	100	100	99	99
WGA-12-4	100	100	100	100	99
WGA-12-6	100	100	100	100	99
WGA-1-9	99	100	100	100	99
WGA-12-16	99	99	99	99	100

Table S2. A total of 248 single-copy marker proteins were identified in genomes of *T*. sp. UBA6158, *T. roseopersicina* DSM 217, *T*. marina 5811, *T*. rosea DSM235, and *T*. sp. LSW.

pfam	single-copy marker protein	pfam	single-copy marker protein	pfam	single-copy marker protein
PF05742.11	TANGO2, Transport	PF00742.18	Homoserine_dh, Homoserine	PF04364.12	DNA_pol3_chi, DNA
PF05299.11	Peptidase_M61, M61	PF05127.13	Helicase_RecD, Helicase	PF01169.18	UPF0016, Uncharacterized
PF01614.17	IclR, Bacterial	PF02219.16	MTHFR, Methylenetetrahydrofolate	PF03710.14	GlnE, Glutamate-ammonia
PF01220.18	DHquinase_II, Dehydroquinase	PF00877.18	NLPC_P60, NlpC/P60	PF07676.11	PD40, WD40-like
PF00829.20	Ribosomal_L21p, Ribosomal	PF01747.16	ATP-sulfurylase, ATP-sulfurylase	PF03449.14	GreA_GreB_N, Transcription
PF13597.5	NRDD, Anaerobic	PF06968.12	BATS, Biotin	PF13436.5	Gly-zipper_OmpA, Glycine-zipper
PF04378.12	RsmJ, Ribosomal	PF06844.10	DUF1244, Protein	PF14701.5	hDGE_amylase, Glycogen
PF05728.11	UPF0227, Uncharacterised	PF07298.10	NnrU, NnrU	PF01219.18	DAGK_prokar, Prokaryotic
PF02113.14	Peptidase_S13, D-Ala-D-Ala	PF01379.19	Porphobil_deam, Porphobilinogen	PF10369.8	ALS_ss_C, Small
PF04079.15	SMC_ScpB, Segregation	PF00398.19	RmaAD, Ribosomal	PF02773.15	S-AdoMet_synt_C, S-adenosylmethionine
PF08547.11	CIA30, Complex	PF03740.12	PdxJ, Pyridoxal	PF06750.12	DiS_P_DiS, Bacterial
PF04257.13	Exonuc_V_gamma, Exodeoxyribonuclease	PF02684.14	LpxB, Lipid-A-disaccharide	PF01520.17	Amidase_3, N-acetylmuramoyl-L-alanine
PF02091.14	tRNA-synt_2e, Glycyl-tRNA	PF14489.5	QueF, QueF-like	PF00708.17	Acylphosphatase, Acylphosphatase
PF00893.18	Multi_Drug_Res, Small	PF04018.12	DUF368, Domain	PF05697.12	Trigger_N, Bacterial
PF04093.11	MreD, rod	PF02578.14	Cu-oxidase_4, Multi-copper	PF00507.18	Oxidored_q4, NADH-ubiquinone/plastoquinone
PF09626.9	DHC, Dihaem	PF04997.11	RNA_pol_Rpb1_1, RNA	PF13307.5	Helicase_C_2, Helicase
PF01029.17	NusB, NusB	PF00698.20	Acyl_transf_1, Acyl	PF02525.16	Flavodoxin_2, Flavodoxin-like
PF12694.6	MoCo_carrier, Putative	PF01564.16	Spermine_synth, Spermine/spermidine	PF02590.16	SPOUT_MTase, Predicted
PF01928.20	CYTH, CYTH	PF04551.13	GcpE, GcpE	PF14849.5	YidC_periplas, YidC
PF02569.14	Pantoate_ligase, Pantoate-beta-alanine	PF02631.15	RecX, RecX	PF03993.11	DUF349, Domain
PF00465.18	Fe-ADH, Iron-containing	PF04379.13	DUF525, ApaG	PF03819.16	MazG, MazG
PF14821.5	Thr_synth_N, Threonine	PF02657.14	SufE, Fe-S	PF01808.17	AICARFT_IMPCHas, AICARFT/IMPCHase
PF04430.13	DUF498, Protein	PF16702.4	DUF5063, Domain	PF02779.23	Transket_pyr, Transketolase,
PF01128.18	IspD, 2-C-methyl-D-erythritol	PF07670.13	Gate, Nucleoside	PF02167.14	Cytochrom_C1, Cytochrome
PF01182.19	Glucosamine_iso, Glucosamine-6-phosphate	PF00318.19	Ribosomal_S2, Ribosomal	PF01252.17	Peptidase_A8, Signal
PF01613.17	Flavin_Reduct, Flavin	PF02616.13	SMC_ScpA, Segregation	PF00468.16	Ribosomal_L34, Ribosomal
PF04186.12	FxsA, FxsA	PF04337.11	DUF480, Protein	PF02464.16	CinA, Competence-damaged
PF02410.14	RsfS, Ribosomal	PF08543.11	Phos_pyr_kin, Phosphomethylpyrimidine	PF05618.10	Zn_protease, Putative
PF06695.10	Sm_multidrug_ex, Putative	PF00406.21	ADK, Adenylate	PF00712.18	DNA_pol3_beta, DNA
PF04952.13	AstE_AspA, Succinylglutamate	PF06508.12	QueC, Queuosine	PF09712.9	PHA_synth_III_E, Poly(R)-hydroxyalkanoic
PF01288.19	HPPK, 7,8-dihydro-6-hydroxymethylpterin-pyrophosphokinase	PF00499.19	Oxidored_q3, NADH-ubiquinone/plastoquinone	PF08379.9	Bact_transglu_N, Bacterial
PF13937.5	DUF4212, Domain	PF16199.4	Radical_SAM_C, Radical_SAM	PF10017.8	Methyltransf_33, Histidine-specific
PF01263.19	Aldose_epim, Aldose	PF02965.16	Met_synt_B12, Vitamin	PF11743.7	DUF3301, Protein
PF05951.12	Peptidase_M15_2, Bacterial	PF14805.5	THDPS_N_2, Tetrahydrodipicolinate	PF03796.14	DnaB_C, DnaB-like
PF09831.8	DUF2058, Uncharacterized	PF01016.18	Ribosomal_L27, Ribosomal	PF04453.13	OstA_C, Organic
PF02643.14	DUF192, Uncharacterized	PF13591.5	MerR_2, MerR	PF13525.5	YfiO, Outer
PF06723.12	MreB_Mbl, MreB/Mbl	PF13372.5	Alginate_exp, Alginate	PF14622.5	Ribonucleas_3_3, Ribonuclease-III-like
PF04303.12	PrpF, PrpF	PF11981.7	DUF3482, Domain	PF03091.14	CutA1, CutA1
PF02538.13	Hydantoinase_B, Hydantoinase	PF01155.18	HypA, Hydrogenase/urease	PF00174.18	Oxidored_molyb, Oxidoreductase
PF07879.10	PHB_acc_N, PHB/PHA	PF01628.20	HrcA, HrcA	PF14255.5	Cys_rich_CPXG, Cysteine-rich
PF00310.20	GATase_2, Glutamine	PF02639.13	DUF188, Uncharacterized	PF01192.21	RNA_pol_Rpb6, RNA
PF01274.21	Malate_synthase, Malate	PF09723.9	Zn-ribbon_8, Zinc	PF00596.20	Aldolase_II, Class
PF04085.13	MreC, rod	PF05899.11	Cupin_3, Protein	PF02190.15	LON_substr_bdg, ATP-dependent
PF00478.24	IMPDH, IMP	PF13417.5	GST_N_3, Glutathione	PF02421.17	FeoB_N, Ferrous
PF00303.18	Thymidylat_synt, Thymidylate	PF01411.18	tRNA-synt_2c, tRNA	PF00885.18	DMRL_synthase, 6,7-dimethyl-8-ribityllumazine
PF01924.15	HypD, Hydrogenase	PF04166.11	PdxA, Pyridoxal	PF01641.17	SelR, SelR
PF05048.12	NosD, Periplasmic	PF01027.19	Bax1-I, Inhibitor	PF07075.10	DUF1343, Protein
PF13116.5	DUF3971, Protein	PF01018.21	GTP1_OBG, GTP1/OBG	PF04930.14	FUN14, FUN14
PF02130.16	UPF0054, Uncharacterized	PF04325.12	DUF465, Protein	PF07497.11	Rho_RNA_bind, Rho
PF13194.5	DUF4010, Domain	PF00463.20	ICL, Isocitrate	PF08753.10	NikR_C, NikR
PF09086.10	DUF1924, Domain	PF06415.12	iPGM_N, BPG-independent	PF09361.9	Phasin_2, Phasin
PF04354.12	ZipA_C, ZipA,	PF07991.11	IlvN, Acetohydroxy	PF05690.13	ThiG, Thiazole
PF02390.16	Methyltransf_4, Putative	PF02547.14	Queuosine_synth, Queuosine	PF00550.24	PP-binding, Phosphopantetheine
PF00731.19	AIRC, AIR	PF08281.11	Sigma70_r4_2, Sigma-70,	PF08003.10	Methyltransf_9, Protein
PF00162.18	PGK, Phosphoglycerate	PF01510.24	Amidase_2, N-acetylmuramoyl-L-alanine	PF02922.17	CBM_48, Carbohydrate-binding
PF06853.11	DUF1249, Protein	PF11067.7	DUF2868, Protein	PF00183.17	HSP90, Hsp90
PF04367.12	DUF502, Protein	PF14437.5	MafB19-deam, MafB19-like	PF03309.13	Pan_kinase, Type
PF01977.15	UbiD, 3-octaprenyl-4-hydroxybenzoate	PF02602.14	HEM4, Uroporphyrinogen-III	PF01116.19	F_bP_aldolase, Fructose-bisphosphate
PF00177.20	Ribosomal_S7, Ribosomal	PF09650.9	PHA_gran_rgn, Putative	PF04127.14	DFP, DNA
PF01960.17	ArgJ, ArgJ	PF10986.7	DUF2796, Protein	PF04359.13	DUF493, Protein
PF14238.5	DUF4340, Domain	PF08676.10	MutL_C, MutL	PF02548.14	Pantoate_transf, Ketopantoate
PF01795.18	Methyltransf_5, MraW	PF06325.12	PrmA, Ribosomal	PF00488.20	MutS_V, MutS
PF01761.19	DHQ_synthase, 3-dehydroquinate	PF01653.17	DNA_ligase_aden, NAD-dependent	PF01955.17	CbiZ, Adenosylcobinamide
PF13500.5	AAA_26, AAA	PF04386.12	SspB, Stringent	PF16123.4	HAGH_C, Hydroxyacylglutathione
PF04204.15	HTS, Homoserine	PF00958.21	GMP_synt_C, GMP	PF04362.13	Iron_traffic, Bacterial
PF00164.24	Ribosom_S12_S23, Ribosomal	PF00120.23	Gln-synt_C, Glutamine	PF00889.18	EF_TS, Elongation
PF01513.20	NAD_kinase, ATP-NAD	PF03602.14	Cons_hypoth95, Conserved	PF04452.13	Methyltrans_RNA, RNA
PF02445.15	NadA, Quinolinate	PF02092.16	tRNA_synt_2f, Glycyl-tRNA	PF00816.20	Histone_HNS, H-NS
PF03755.12	YicC_N, YicC-like	PF12631.6	MnmE_helical, MnmE	PF01406.18	tRNA-synt_1e, tRNA
PF09382.9	RQC, RQC	PF12447.7	DUF3683, Protein	PF03186.12	CobD_Cbib, CobD/Cbib
PF00490.20	ALAD, Delta-aminolevulinic	PF01149.23	Fapy_DNA_glyco, Formamidopyrimidine-DNA	PF11042.7	DUF2750, Protein
PF01142.17	TruD, tRNA	PF00939.18	Na_sulph_symp, Sodium:sulfate	PF05872.11	DUF853, Bacterial
PF13598.5	DUF4139, Domain	PF01075.16	Glyco_transf_9, Glycosyltransferase	PF14815.5	NUDIX_4, NUDIX
PF00186.18	DHFR_1, Dihydrofolate	PF11736.7	DUF3299, Protein	PF02347.15	GDC-P, Glycine
PF07264.10	EI24, Etoposide-induced	PF03649.12	UPF0014, Uncharacterised	PF02574.15	S-methyl_trans, Homocysteine
PF00033.18	Cytochrome_B, Cytochrome	PF01784.17	NIF3, NIF3	PF01597.18	GCV_H, Glycine
PF09834.8	DUF2061, Predicted	PF03881.13	Fructosamin_kin, Fructosamine	PF02777.17	Sod_Fe_C, Iron/manganese
PF13202.5	EF-hand_5, EF	PF04608.12	PgpA, Phosphatidylglycerophosphatase	PF01947.15	DUF98, Protein
PF04976.11	DmsC, DMSO	PF00227.25	Proteasome, Proteasome		
PF12263.7	DUF3611, Protein	PF00625.20	Guanylate_kin, Guanylate		

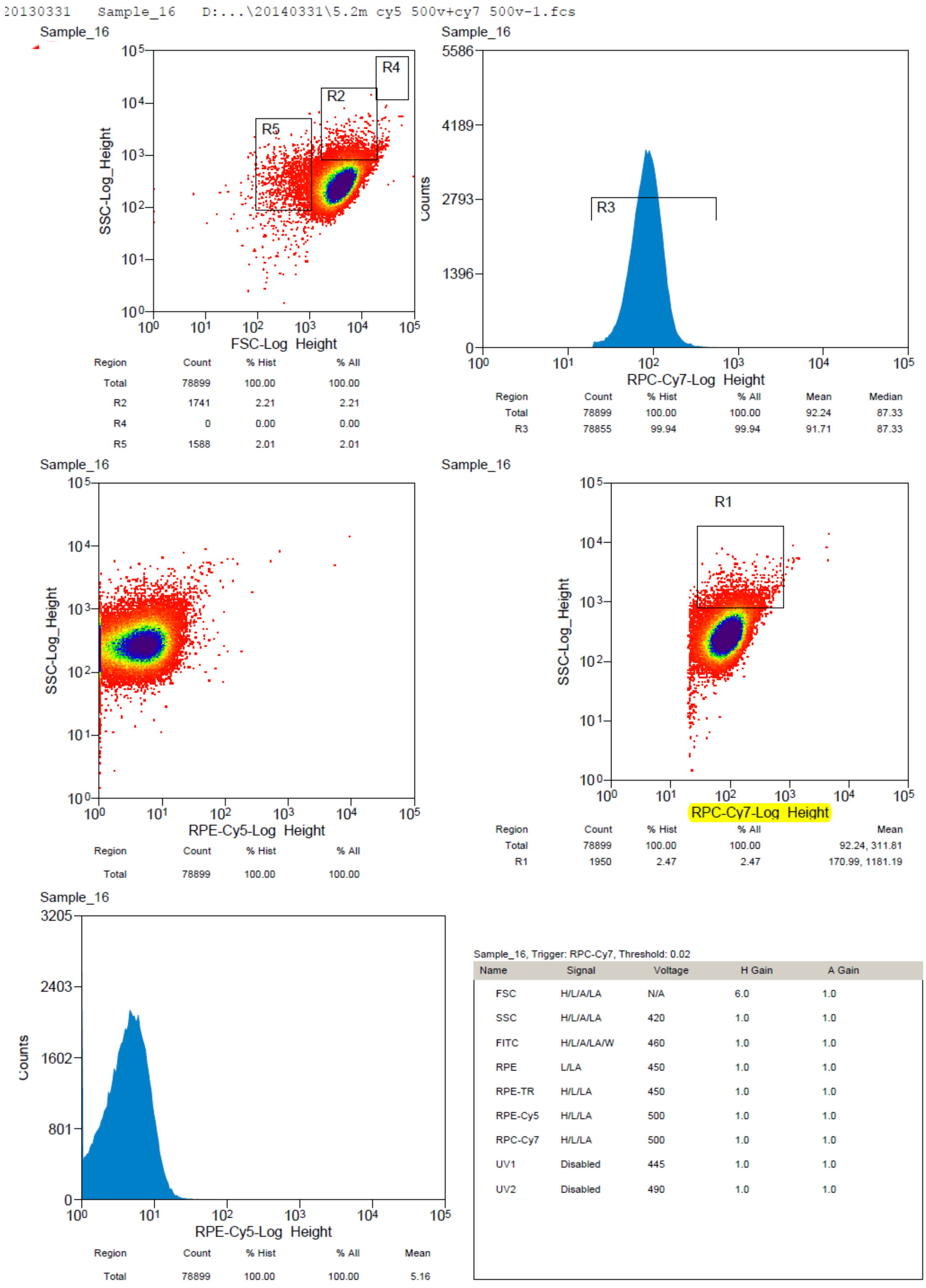

Figure S1. Flow cytometric identification of *Thiocapsa* sp. LSW using **Beckman Coulter MoFlo XDP cell sorter.**

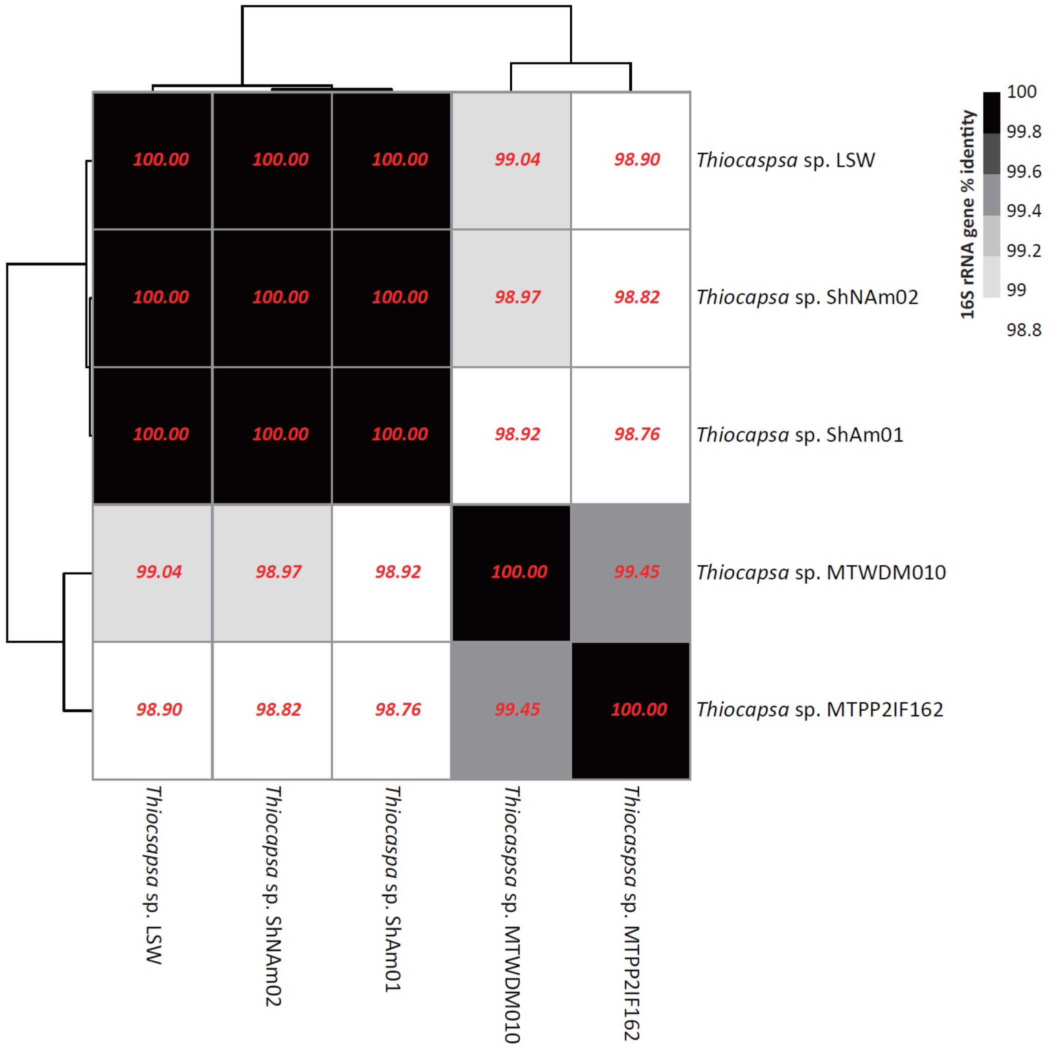

Figure S2 A heatmap based on the 16S rRNA gene identity (%) between the closest relatives of Thiocapsa sp. in Fig 1a.

(a)

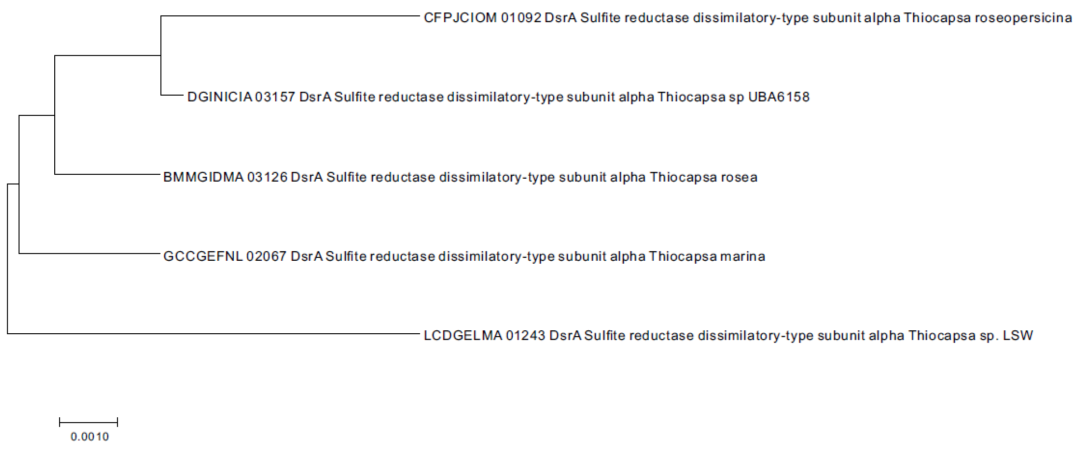

(b)

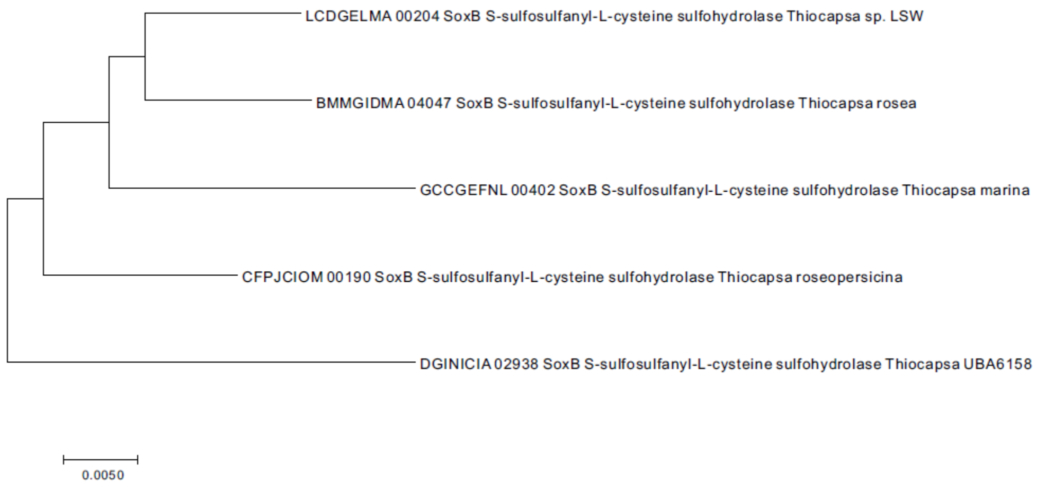

Figure S3 Phylogenetic tree of Thiocapsa species based on (a) *dsrA* (b) *soxB* genes were constructed using the maximum-likelihood method with 1000 bootstraps.
